# Incorporating Continuous Mammographic Density Into the BOADICEA Breast Cancer Risk Prediction Model

**DOI:** 10.1200/PO-25-00203

**Published:** 2025-09-26

**Authors:** Lorenzo Ficorella, Mikael Eriksson, Kamila Czene, Goska Leslie, Xin Yang, Tim Carver, Adam E. Stokes, Douglas F. Easton, Per Hall, Antonis C. Antoniou

**Affiliations:** ^1^ Centre for Cancer Genetic Epidemiology, Department of Public Health and Primary Care, University of Cambridge, Cambridge, United Kingdom; ^2^ Department of Medical Epidemiology and Biostatistics, Karolinska Institutet, Stockholm, Sweden; ^3^ Centre for Cancer Genetic Epidemiology, Department of Oncology, University of Cambridge, Cambridge, United Kingdom; ^4^ Department of Oncology, Södersjukhuset, Stockholm, Sweden

## Abstract

**PURPOSE:**

Breast and Ovarian Analysis of Disease Incidence and Carrier Estimation Algorithm (BOADICEA v7) predicts future breast cancer (BC) risk using data on cancer family history (FH), genetic markers, questionnaire-based risk factors, and mammographic density (MD) measured using the four-category Breast Imaging Reporting and Data System (BIRADS) classification. However, BIRADS requires manual reading, which is impractical on a large scale and may cause information loss. We extended BOADICEA to incorporate continuous MD measurements, calculated using the automated Volpara and STRATUS tools.

**METHODS:**

We used data from the Karolinska Mammography Project for Risk Prediction of Breast Cancer cohort (60,276 participants; 1,167 incident BC). Associations between MD measurements and BC risk were estimated in a randomly selected training subset (two thirds of the data set). Percent MD residuals were calculated after regressing on age at mammography and BMI. Hazard ratios (HRs) were estimated using a Cox proportional hazards model, adjusting for FH and BOADICEA risk factors, and were incorporated into BOADICEA. The remaining one third of the cohort was used to assess the performance of the extended BOADICEA (v7.2) in predicting 5-year risks.

**RESULTS:**

The BC HRs per standard deviation of residual STRATUS density were estimated to be 1.48 (95% CI, 1.33 to 1.64) and 1.41 (95% CI, 1.27 to 1.56) for pre- and postmenopausal women, respectively. The corresponding estimates for Volpara density were 1.27 (95% CI, 1.15 to 1.40) and 1.38 (95% CI, 1.25 to 1.54). The extended BOADICEA showed improved discrimination in the testing data set over using BIRADS, with a 1%-4% increase in AUC across different combinations of risk factors. On the basis of 5-year BC risk with MD as the sole input, approximately 11% of the women were reclassified into lower risk categories and 18% into higher risk categories using the extended model.

**CONCLUSION:**

Incorporating continuous MD measurements into BOADICEA enhances BC risk stratification and facilitates the use of automated MD measures for risk prediction.

## Graphical abstract

**Figure:**
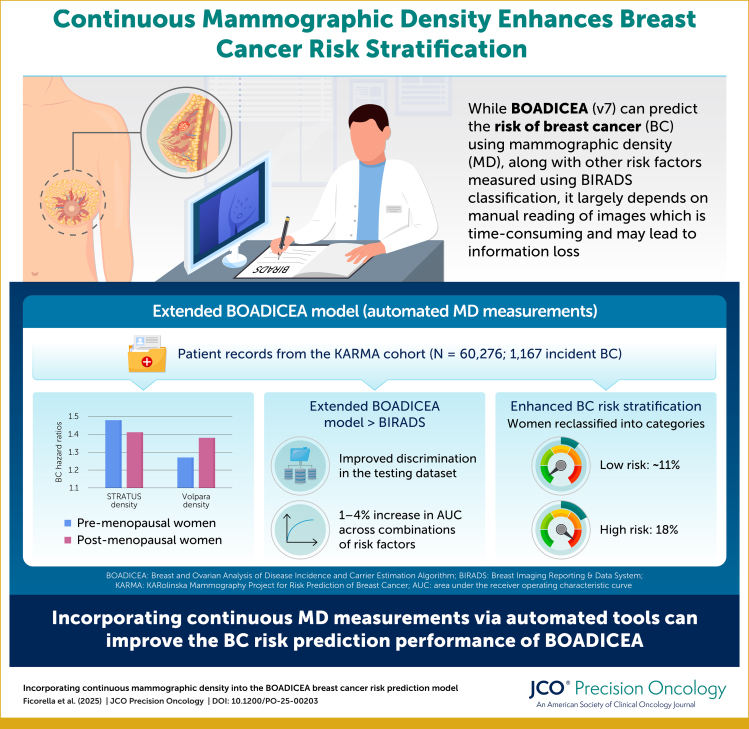

## INTRODUCTION

Breast cancer (BC) is the most common cancer in women.^1,2^ Early detection through screening and risk reduction through preventive medication or surgery are available,^3-5^ but they may be associated with overdiagnosis, overtreatment, or other side effects. BC risk prediction models can help targeting such approaches to those likely to benefit, thus optimizing resource allocation.^6^

**CONTEXT**

**Key Objective**
To extend Breast and Ovarian Analysis of Disease Incidence and Carrier Estimation Algorithm (BOADICEA) breast cancer (BC) risk prediction model (implemented in the CanRisk tool) so that it incorporates continuous mammographic density (MD) measurements calculated using the automated Volpara and STRATUS tools.
**Knowledge Generated**
The extended BOADICEA showed improved discrimination, with a 1%-4% increase in AUC across different combinations of risk factors (3%-4% when using the full model). On the basis of the 5-year BC risk with MD as the sole input, approximately 29% of the women were reclassified into different risk categories using the extended model.
**Relevance**
This extension enhances BC risk stratification and facilitates the use of automated MD measures for risk prediction in clinical practice.

Breast and Ovarian Analysis of Disease Incidence and Carrier Estimation Algorithm (BOADICEA) BC risk model v7,^7-9^ implemented in the CanRisk tool,^10^ can be used to predict BC risk using data on a combination of factors including cancer family history (FH), pathogenic genetic variants, polygenic scores (PGS), and lifestyle, hormonal, and reproductive risk factors (referred to here as questionnaire-based risk factors [QRFs]). BOADICEA also includes mammographic density (MD),^8,11^ using the Breast Imaging Reporting and Data System (BIRADS) density categories.^12^

MD measures the amount of stromal and epithelial tissue in the breast, which appear as brighter regions in mammograms compared with adipose tissue.^13^ MD is usually measured in terms of the proportion of dense breast. Higher MD is associated with increased BC risk,^14,15^ and MD has been included in several BC risk models (eg, Lee et al,^11^ Eriksson et al,^16^ Brentnall et al^17^). MD is influenced by many other factors, including age, BMI, and menopausal status, and was found to vary by ethnicity.^18-20^ The most widely used method for evaluating MD is the four-category BIRADS.^12^ BIRADS relies on manual reading of images. However, this is time-consuming and is prone to inter- and intraoperator variability.^21,22^ Advances in image-based analysis have led to fully automated tools for measuring MD (eg, STRATUS,^23^ Volpara,^24^ Quantra^25^). These produce MD estimates on a continuous scale, expressed as a percentage of either image area (percent mammographic density [PMD]) or breast volume (percent volumetric density [PVD]). STRATUS calculates PMD and can be applied to processed images; Volpara calculates PVD and requires raw images.

Using fully automated tools in clinical practice can be more cost effective at population scale and improve consistency in MD assessment. Moreover, BC risk varies continuously with MD, and use of categorical measures such as BIRADS results in information loss. Continuous MD measures have been shown to be stronger risk predictors than categorical measures, for example, in terms of log-odds per adjusted standard deviation (OPERA).^26-28^

Here, we used data from a large prospective cohort of women enrolled in the mammographic screening program in Sweden to derive risk estimates for continuous MD measured by Volpara and STRATUS software. We extended BOADICEA by incorporating continuous MD into the BC risk model (v7.2) and validated the resulting model in an independent subset of the cohort.

## METHODS

### Study Population

The Karolinska Mammography Project for Risk Prediction of Breast Cancer (KARMA) cohort enrolled women from Sweden (age range, 40-74) undergoing mammographic screening between 2011 and 2013^29^; participants were followed until December 2019. Study participants provided self-reported first-degree cancer FH and data on QRFs. The first mammogram at study entry was used to measure MD using STRATUS^23^ and Volpara,^30^ and to derive equivalent BIRADS categorical scores computationally (cBIRADS^31^). Data on cancer diagnoses and deaths were obtained from linkages to health care registers. Data on the 313-SNP PGS^32^ were available for about a quarter of the participants: half of the BC incident cases and a randomly selected subset of unaffected women from the entire cohort.^33^ Further details can be found in the Data Supplement.

Analysis was restricted to women who were unaffected with BC at recruitment, had not had risk-reducing mastectomy, and for whom MD measurements were available. To exclude prevalent cancers, women diagnosed with cancer (or otherwise censored) within 1 year from study entry were also excluded. The eligible cohort was split randomly into a training (two thirds of the data set) and a testing set (one third of the data set), preserving the same case-to-control ratio in both sets. Missing data were imputed using Multivariate Imputation by Chained Equations^34^ (Data Supplement).

### Ethical Approval

KARMA study was approved by the ethics review board at Karolinska Institutet. All participants provided written informed consent to participate in the study before taking part.

### Statistical Methods

#### Residuals and Normalization

A key assumption of the model is that, by regressing out the effect of age, the standardized PMD/PVD can then be considered as a fixed covariate. Therefore, we first calculated the residuals of PMD (STRATUS) and PVD (Volpara) after regressing on age at entry and BMI (without interaction) using linear regression (*lm* function, R^35^). Residuals were then transformed through a two-parameter Box-Cox transformation to obtain a Gaussian distribution and then further standardized to obtain a standard normal distribution (Data Supplement, Table S1). Separately, residuals of PMD and PVD were calculated after regressing on age at entry only; residuals were then transformed and standardized in the same way (Data Supplement, Table S1).

#### Associations with BC Risk

Associations between the standardized PMD and PVD residuals and BC risk were evaluated in the training data set using a Cox proportional hazard model to estimate the hazard ratio (HR) for BC per standard deviation (SD) of the standardized residual PMD/PVD. Participants were followed from study entry until the first event among: cancer (in situ/invasive BC, other cancer), prophylactic mastectomy, death, 80 years of age, or last follow-up date. Women were considered affected only if they developed invasive BC.

The Cox regression models (*coxph* function, R survival package^36^) were adjusted for FH and for the additive effects (on log-scale) of all other BOADICEA QRFs^11^ (Table 1). FH was included as categorical variable based on the number of affected first-degree relatives (0, 1, ≥2). An interaction term between menopausal status and standardized residual PMD/PVD was added as a covariate, allowing separate HR estimates for premenopausal and postmenopausal women to be derived.

**TABLE 1.** HR Estimates per SD of the Standardized STRATUS PMD and Volpara PVD Residuals; Obtained by Regressing on Age and BMI, and Then by Adjusting for FH and QRFs (BMI excluded). QRFs Considered: Age at First Live Birth, Age at Menarche, Age at Menopause (if applicable), Alcohol Consumption, Height, Parity, Use of Oral Contraceptives, Use of Hormone Replacement Therapy

HR Estimation Options	STRATUS			Volpara		
	Unknown Status	Premenopausal	Postmenopausal	Unknown Status	Premenopausal	Postmenopausal
Unadjusted for PGS	1.44 (1.34-1.55)	1.48 (1.33-1.65)	1.41 (1.27-1.56)	1.32 (1.23-1.42)	1.27 (1.15-1.41)	1.38 (1.25-1.54)
Adjusted for PGS	1.41 (1.31-1.52)	1.45 (1.30-1.62)	1.38 (1.25-1.53)	1.31 (1.22-1.41)	1.26 (1.13-1.39)	1.37 (1.23-1.53)

Abbreviations: FH, family history; HR, hazard ratio; PGS, polygenic scores; PMD, percent mammographic density; PVD, percent volumetric density; QRF, questionnaire-based risk factor; SD, standard deviation.

Analyses were repeated on the subcohort of participants with PGS using a weighted Cox regression approach; each study participant was weighted by the inverse of their probability of inclusion in the subcohort with genotype data (Data Supplement). To accommodate the fact that BMI may not always be available (in clinical practice or in other data sets), additional analyses were conducted, based on residuals of PMD and PVD after regressing on age at entry only (Data Supplement).

### Model Implementation

The resulting BC HR estimates for residual STRATUS PMD and Volpara PVD were included in BOADICEA v7 by adding the log(HR) to the linear predictor, thus providing two alternative options for inputting MD in addition to BIRADS. For this, we adopted the methodology previously developed for incorporating continuous QRFs into BOADICEA^7^ (Data Supplement). The calculation of the residuals and normalization were integrated within the model (using the formulae derived) so that clinical users only need to input information on PMD or PVD. Separately, we also updated the model to use age-specific distributions (before and after 50 years) for BIRADS categories. For this, parameters were derived from the literature^8^; the KARMA cohort was solely used for the validation stage (Data Supplement). The extended model is referred to as BOADICEA v7.2.

### Model Validation

We used the BOADICEA BC risk models (v7 and v7.2) to predict BC risk in the testing data set, starting from 1 year after the age at baseline^9^ using Swedish age-specific and calendar period–specific population cancer incidences. BC risk was calculated at 5 years or the censored age (whichever occurred first); women were considered as unaffected if they developed BC after the predicted 5-year interval.

We investigated the reclassification between risk categories when predicting 5-year BC risks using the two models. Four risk categories were considered, as reported in the literature for other risk prediction models^37^: below 1%, between 1% and <1.67%, between 1.67% and <3%, and above 3%. To investigate model behavior in low-risk subpopulations, the lowest category was further split at 0.35% (approximately half the 5-year population risk for a 40-year-old woman), thus yielding five risk categories. Risks were predicted using MD measures only (to highlight the effect of the different MD reporting methods) or the full available input. Absolute Net Reclassification Improvement index (NRI^38^, range [−2 to 2]) was also calculated.

We assessed the model performance in terms of calibration (eg, using calibration slope by regressing the BC status on the log-odds of the predicted risk) and discriminative ability (AUC, area under the receiver operating characteristic curve) across different risk factor combinations (Data Supplement).

## RESULTS

After exclusions (Data Supplement), the final data set (Data Supplement, Tables S2 and S3) included 60,276 women (age 25-74 years) unaffected at study entry, 1,167 of whom developed invasive BC (mean/median follow-up of 7.6 years). Of these, 981 women developed invasive BC in the 5-year risk prediction horizon used in the validation stage.

The training set (Data Supplement, Table S4) comprised 40,184 participants and 778 incident cases of invasive BCs (658 within the 5-year period), whereas the testing set (Data Supplement, Table S5) comprised 20,092 participants and 389 incident cases. There were no significant differences in the risk factor distributions between the training and testing sets (all *P* values > .2); approximately a quarter of participants in each set had PGS data. The Data Supplement and Figure S1 show the distribution of density residuals after regressing on age at entry and BMI.

### Associations With BC Risk

Table 1 shows the estimated HRs per SD of the standardized STRATUS PMD and Volpara PVD residuals after regressing on age and BMI. When adjusting for all QRFs in BOADICEA except PGS, the HRs per SD of residual STRATUS PMD were estimated to be 1.48 (95% CI, 1.33 to 1.64) and 1.41 (95% CI, 1.27 to 1.56) for pre- and postmenopausal women, respectively. The corresponding HR estimates per SD of residual Volpara PVD density were 1.27 (95% CI, 1.15 to 1.40) and 1.38 (95% CI, 1.25 to 1.54) for pre- and postmenopausal women, respectively. In analyses based on residuals after regression on age only, the HRs were all slightly (7%-9%) larger (Data Supplement, Table S6).

In the training subcohort with PGS data, there was a well-fitting linear relationship between HRs estimated with and without adjusting for PGS (Data Supplement). Using this relationship, we calculated the HRs per SD that would have been obtained by adjusting for PGS on the whole training set (Table 1 and Data Supplement, Table S6). Adjusting for the PGS resulted in an attenuation in the HR estimates by 2% for STRATUS and 1% for Volpara.

### Augmented BOADICEA

BOADICEA v7 was extended by including the HR estimates summarized in Table 1 and the Data Supplement (Table S6), the regression parameters provided in the Data Supplement (Table S1), and published age-specific BIRADS distributions^8^; all other model parameters remained unaltered.

As a result, BOADICEA v7.2 accepts BIRADS categories, STRATUS PMD, and Volpara PVD as inputs. Model functioning, based on the input provided, is described in the Data Supplement.

### Risk Distributions

#### Predicted Range of Risks (BOADICEA v7.2)

**FIG 1.**
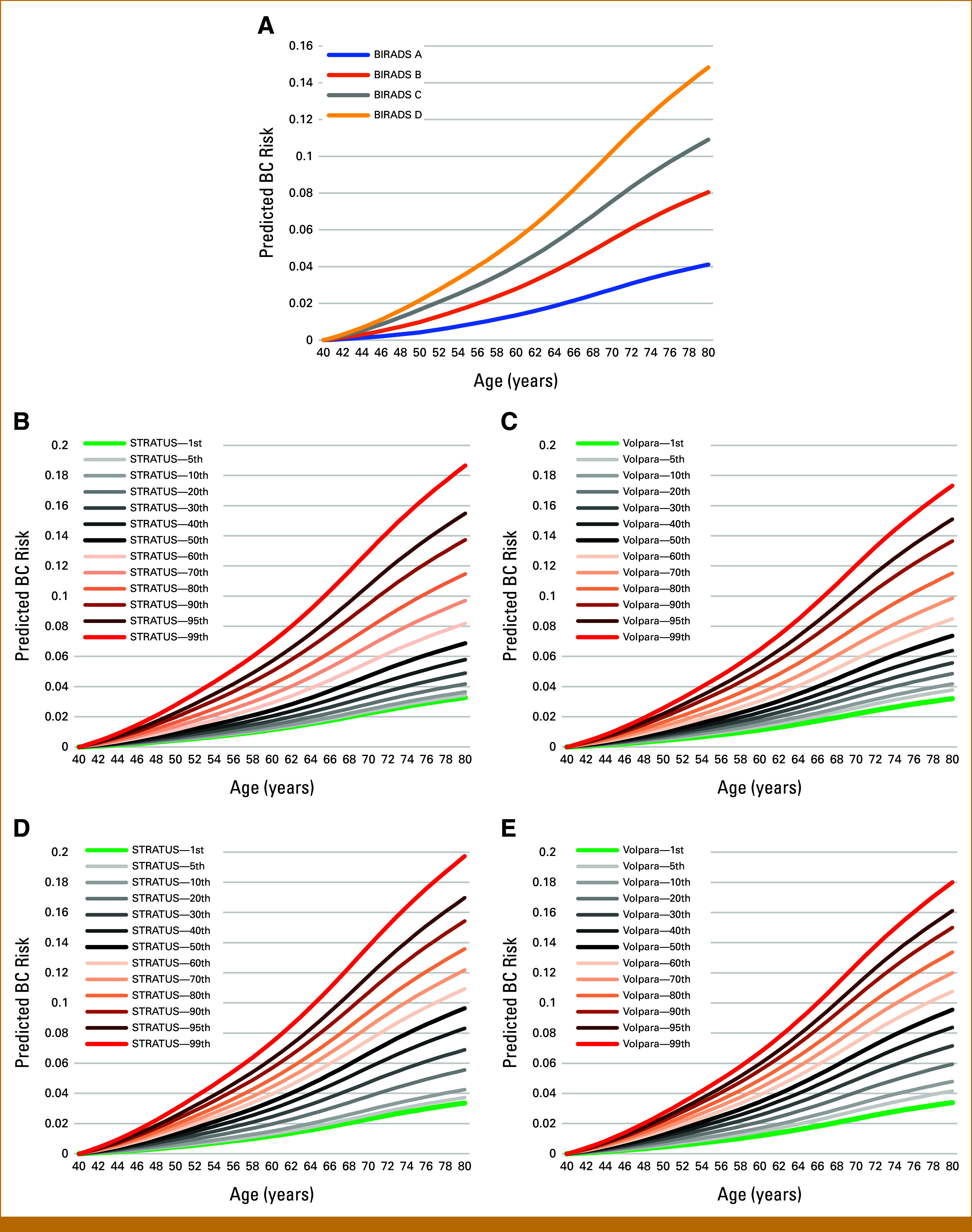
Predicted age-specific BC risk to age 80 years for a 40-year woman (born in 1985) using the augmented model. Only MD assumed as input information. (A) For the four different BIRADS categories; (B) for percentiles of the STRATUS PMD distribution in KARMA; (C) for percentiles of the Volpara PVD distribution in KARMA; (D) for percentiles of the STRATUS PMD distribution on women <50 years in KARMA; (E) for percentiles of the Volpara PVD distribution on women <50 years in KARMA. Percentiles considered: 1st, 5th, 10th…90th, 95th, 99th. BC, breast cancer; BIRADS, Breast Imaging Reporting and Data System; KARMA, Karolinska Mammography Project for Risk Prediction of Breast Cancer; MD, mammographic density; PMD, percent mammographic density; PVD, percent volumetric density.

Figure 1 shows the predicted BC risks for a hypothetical 40-year-old woman, calculated at each year up to 80. The range of predicted risks (defined as the 1st to 99th percentile) were similar for STRATUS PMD and Volpara PVD; both were wider than the range of risks obtained using the BIRADS categories. For example, the predicted remaining lifetime BC risks (ie, risk to 80 years) ranged from 0.041 to 0.148 when using BIRADS, from 0.033 to 0.187 when using STRATUS, and from 0.032 to 0.173 when using Volpara. The predicted BC risks by BMI category and MD measurement method are shown in the Data Supplement (Figs S2-S4).

#### Predicted 5-Year Risks in the KARMA Testing Set

**FIG 2.**
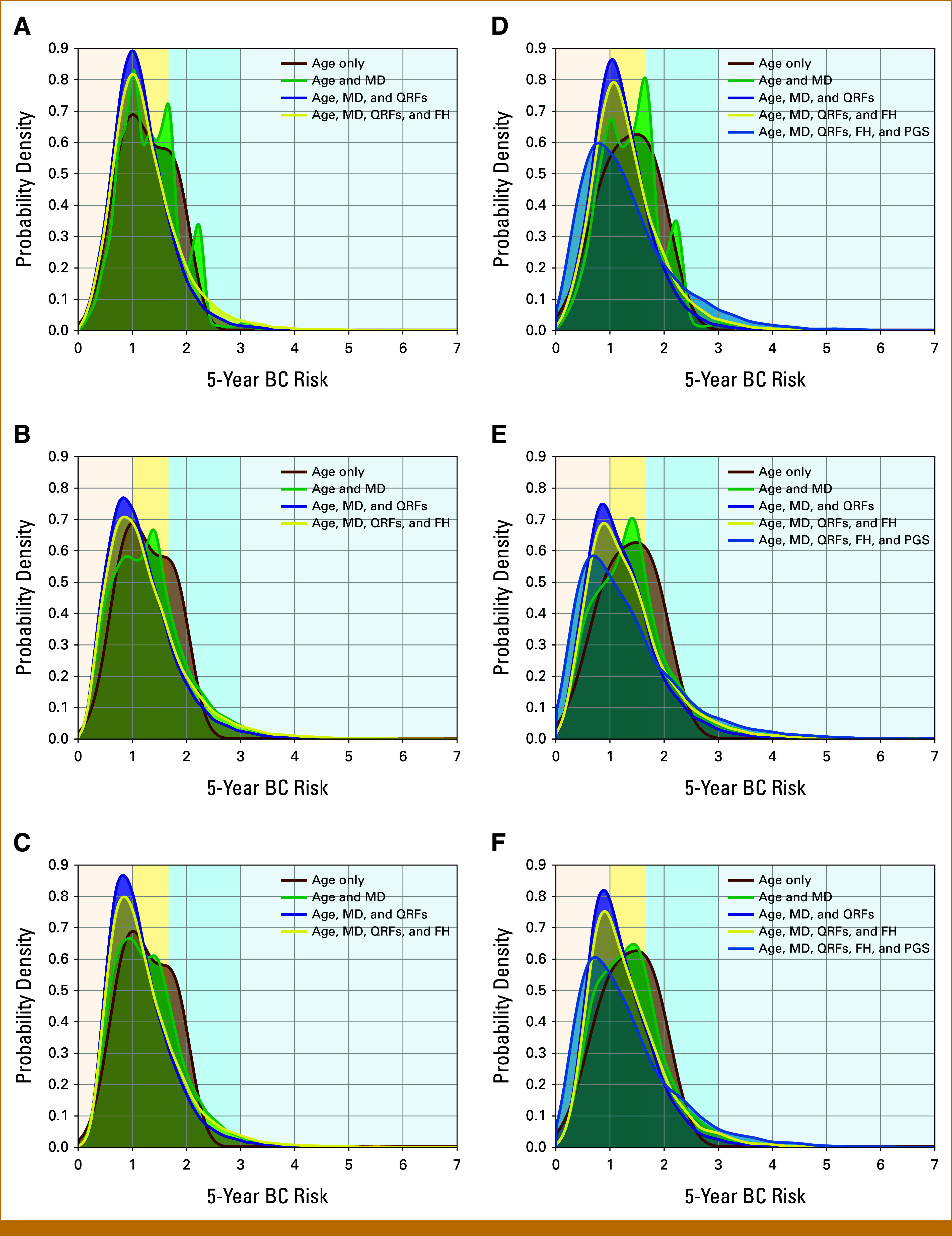
Predicted 5-year BC risk, based on different combinations of risk predictors: (1) age, MD, QRFs, and FH for all women in the testing data set (A-C); (2) age, MD, QRFs, FH, and PGS for the subcohort of women with PGS data (D-F). All figures show the probability density against the absolute risk: (A and D) using BIRADS categories; (B and E) using STRATUS PMD measures; (C and F) using Volpara PVD measures. The backgrounds of the graphs are shaded to indicate four 5-year BC risk categories: <1% (light yellow); 1%-1.67% (yellow); 1.67%-3% (blue); ≥3% (light blue). BC, breast cancer; BIRADS, Breast Imaging Reporting and Data System; FH, family history; MD, mammographic density; PGS, polygenic scores; PMD, percent mammographic density; PVD, percent volumetric density; QRF, questionnaire-based risk factor.

The distribution of predicted 5-year risks in the testing data set is shown in Figure 2 and the Data Supplement (Figs S5 and S6). When using BIRADS, the distributions showed multiple peaks corresponding to the different categories. The risk distributions using STRATUS or Volpara appeared bimodal, reflecting the different HRs per SD for premenopausal and postmenopausal women. However, the distributions became unimodal when additional predictors were included.

The predicted risk distributions were slightly wider when using STRATUS and Volpara instead of BIRADS. This resulted in differences in risk classification; Table 2 shows the corresponding proportions of women in the different risk categories. When using MD only, the proportion of women in both the highest and lowest risk categories increased when using continuous MD rather than BIRADS categories. When using all risk factors (MD, FH, QRFs and PGS), the proportion of women in the highest risk category (≥3%) increased from 0.03 (original model) to 0.04 (Volpara) and 0.05 (STRATUS); however, the proportion in the lowest risk category (<0.35%) increased only when using STRATUS (from 0.084 to 0.098).

**TABLE 2.** Proportion of Women Classified in Four Risk Categories (thresholds derived from Tice et al, 2015^37^); 5-Year BC Risks Calculated Using the Original BOADICEA v7 Model and the Augmented Model, Using BIRADS Categories, STRATUS PMD, and Volpara PVD as MD Inputs. Risk Distributions Obtained With Models (1) Considering MD Only and (2) Considering MD, FH, QRFs, and PGS; all Models Include Age by Default

MD Methods and Subsets Considered	Distributions, Considering MD Only					
	<0.35%	0.35%-1%	<1%	[1%-1.67%)	[1.67%-3%)	≥3%
All women						
BOADICEA v7	0.009	0.296	0.304	0.549	0.146	0.000
New model (BIRADS)	0.009	0.285	0.293	0.450	0.250	0.007
New model (STRATUS)	0.030	0.324	0.354	0.399	0.224	0.022
New model (Volpara)	0.015	0.339	0.354	0.408	0.224	0.015
Unaffected only						
BOADICEA v7	0.009	0.298	0.307	0.549	0.145	0.000
New model (BIRADS)	0.009	0.287	0.295	0.450	0.249	0.006
New model (STRATUS)	0.030	0.327	0.357	0.399	0.222	0.022
New model (Volpara)	0.015	0.341	0.356	0.408	0.222	0.014
Affected only						
BOADICEA v7	0.004	0.144	0.148	0.583	0.269	0.000
New model (BIRADS)	0.004	0.140	0.144	0.443	0.395	0.018
New model (STRATUS)	0.018	0.146	0.164	0.413	0.392	0.031
New model (Volpara)	0.004	0.199	0.203	0.375	0.388	0.034

MD Methods and Subsets Considered	Distributions, Considering Full Model					
	<0.35%	0.35%-1%	<1%	[1%-1.67%)	[1.67%-3%)	≥3%
All women						
BOADICEA v7	0.084	0.425	0.508	0.294	0.165	0.033
New model (BIRADS)	0.076	0.384	0.459	0.304	0.191	0.046
New model (STRATUS)	0.098	0.397	0.495	0.272	0.183	0.050
New model (Volpara)	0.083	0.414	0.497	0.277	0.183	0.043
Unaffected only						
BOADICEA v7	0.084	0.427	0.512	0.293	0.163	0.033
New model (BIRADS)	0.076	0.386	0.463	0.303	0.189	0.046
New model (STRATUS)	0.099	0.400	0.499	0.271	0.181	0.049
New model (Volpara)	0.084	0.417	0.501	0.276	0.180	0.043
Affected only						
BOADICEA v7	0.020	0.227	0.247	0.372	0.301	0.080
New model (BIRADS)	0.017	0.188	0.205	0.351	0.345	0.098
New model (STRATUS)	0.013	0.199	0.212	0.309	0.347	0.132
New model (Volpara)	0.013	0.208	0.222	0.315	0.357	0.106

Abbreviations: BC, breast cancer; BIRADS, Breast Imaging Reporting and Data System; BOADICEA, Breast and Ovarian Analysis of Disease Incidence and Carrier Estimation Algorithm; FH, family history; MD, mammographic density; PGS, polygenic scores; PMD, percent mammographic density; PVD, percent volumetric density; QRF, questionnaire-based risk factor.

When using all risk factors (MD, FH, QRFs, and PGS) and comparing with the results obtained using BOADICEA v7, 8%-10% of women were reclassified to lower risk categories and 14% of women to higher risk categories when STRATUS or Volpara was used (Data Supplement, Table S7), with absolute NRIs of 0.123 and 0.087, respectively.

### Model Validation

**FIG 3.**
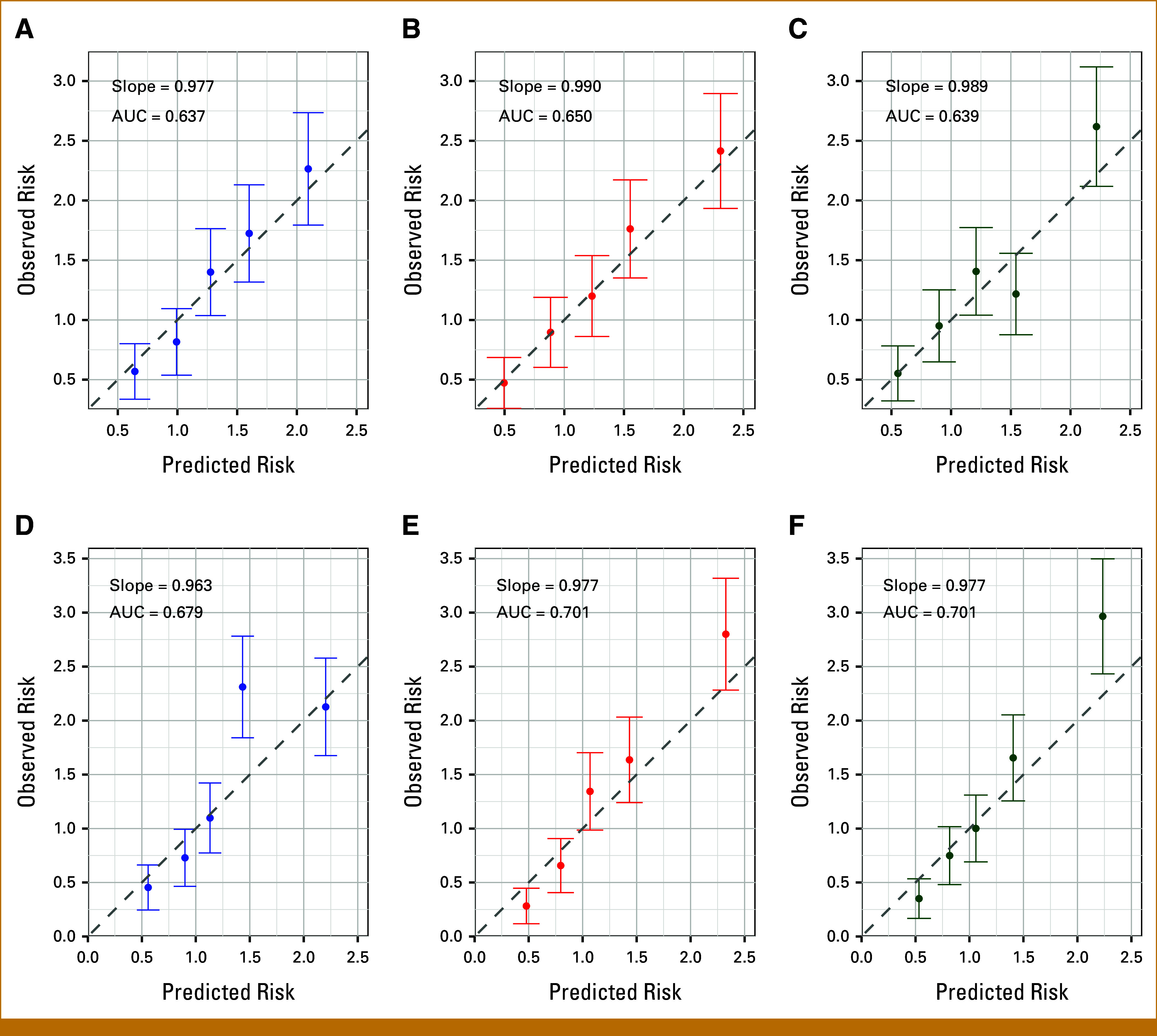
Calibration of the absolute predicted 5-year BC risk, showing the observed and expected risks by quintiles of the predicted risks. Each dot represents the mean observed and predicted risk in the quintile; the bars show the 95% CIs for the observed risks (calculated assuming a Poisson distribution). (A-C) Risks calculated on the entire testing data set, using age and MD as inputs. (D-F) Risks calculated on the testing subset containing PGS data, using all available inputs (age, MD, QRFs, FH and PGS). (A and D) BIRADS; (B and E) STRATUS; (C and F) Volpara. BC, breast cancer; BIRADS, Breast Imaging Reporting and Data System; FH, family history; MD, mammographic density; PGS, polygenic scores; QRF, questionnaire-based risk factor.

The extended model was well calibrated for predicting the 5-year BC risk for all combinations of predictors, showing a slight improvement in the calibration slope compared with BOADICEA v7 (Table 3). For example, when using the full input, the calibration slope was 0.98 (95% CI, 0.98 to 0.99) for STRATUS, Volpara, and age-specific BIRADS compared with 0.97 (95% CI, 0.96 to 0.98) for BOADICEA v7. The corresponding models were also well calibrated in quintiles of predicted risk (Fig 3).

**TABLE 3.** Calibration Slope and AUC Calculated on Risks Predicted by the Original BOADICEA v7 Model and the New Model, Using BIRADS, STRATUS, and Volpara as MD Inputs

Inputs Combination	Calibration Slope on the Testing Set, Full Cohort			
	BOADICEA v7	BIRADS	STRATUS	Volpara
Age + MD	0.98 (0.97 to 0.99)	1.00 (0.99 to 1.00)	0.99 (0.98 to 1.00)	0.99 (0.98 to 1.00)
Age + MD + QRFs	0.95 (0.94 to 0.96)	0.97 (0.96 to 0.98)	0.96 (0.96 to 0.97)	0.96 (0.95 to 0.97)
Age + MD + FH	0.99 (0.98 to 1.00)	1.01 (1.00 to 1.02)	1.01 (1.00 to 1.02)	1.01 (1.00 to 1.01)
Age + MD + QRFs + FH	0.97 (0.96 to 0.98)	0.98 (0.98 to 0.99)	0.98 (0.97 to 0.99)	0.98 (0.97 to 0.99)

Inputs Combination	Calibration Slope on the Testing Set, Subcohort With PGS			
	BOADICEA v7	BIRADS	STRATUS	Volpara
Age + MD	0.98 (0.97 to 0.99)	0.98 (0.97 to 0.99)	1.00 (0.99 to 1.01)	0.99 (0.98 to 1.00)
Age + MD + QRFs	0.95 (0.94 to 0.96)	0.95 (0.94 to 0.96)	0.97 (0.96 to 0.98)	0.96 (0.95 to 0.97)
Age + MD + FH	1.00 (0.99 to 1.01)	1.00 (0.99 to 1.00)	1.02 (1.01 to 1.02)	1.01 (1.00 to 1.02)
Age + MD + QRFs + FH	0.97 (0.96 to 0.98)	0.97 (0.96 to 0.98)	0.98 (0.98 to 0.99)	0.98 (0.97 to 0.99)
Age + MD + QRFs + FH + PGS	0.96 (0.96 to 0.97)	0.96 (0.95 to 0.97)	0.98 (0.97 to 0.99)	0.98 (0.97 to 0.99)

Inputs Combination	AUC on the Testing Set, Full Cohort			
	BOADICEA v7	BIRADS	STRATUS	Volpara
Age + MD	0.64 (0.63 to 0.65)	0.64 (0.63 to 0.65)	0.65 (0.64 to 0.66)	0.64 (0.63 to 0.65)
Age + MD + QRFs	0.65 (0.64 to 0.66)	0.65 (0.64 to 0.66)	0.67 (0.66 to 0.68)	0.66 (0.66 to 0.67)
Age + MD + FH	0.64 (0.63 to 0.65)	0.64 (0.63 to 0.66)	0.66 (0.65 to 0.67)	0.65 (0.64 to 0.66)
Age + MD + QRFs + FH	0.66 (0.65 to 0.67)	0.66 (0.65 to 0.67)	0.68 (0.67 to 0.69)	0.67 (0.66 to 0.68)

Inputs Combination	AUC on the Testing Set, Subcohort With PGS			
	BOADICEA v7	BIRADS	STRATUS	Volpara
Age + MD	0.64 (0.63 to 0.66)	0.65 (0.63 to 0.66)	0.66 (0.64 to 0.67)	0.66 (0.64 to 0.67)
Age + MD + QRFs	0.65 (0.64 to 0.66)	0.66 (0.65 to 0.67)	0.68 (0.67 to 0.69)	0.69 (0.68 to 0.70)
Age + MD + FH	0.64 (0.63 to 0.66)	0.65 (0.64 to 0.66)	0.66 (0.64 to 0.67)	0.66 (0.64 to 0.67)
Age + MD + QRFs + FH	0.66 (0.65 to 0.67)	0.67 (0.65 to 0.68)	0.69 (0.68 to 0.70)	0.69 (0.68 to 0.70)
Age + MD + QRFs + FH + PGS	0.68 (0.67 to 0.69)	0.68 (0.67 to 0.70)	0.70 (0.69 to 0.71)	0.70 (0.69 to 0.71)

NOTE. Multiple inputs combinations tested; 95% CI reported in parentheses.

Abbreviations: BIRADS, Breast Imaging Reporting and Data System; BOADICEA, Breast and Ovarian Analysis of Disease Incidence and Carrier Estimation Algorithm; FH, family history; MD, mammographic density; QRF, questionnaire-based risk factor; PGS, polygenic scores.

There was an increase in the AUC under the extended models using STRATUS or Volpara, compared with BOADICEA v7, with improvements of 1% to 4% depending on the combinations of predictors used (Table 3 and Data Supplement, Table S8). For example, when using all predictors including PGS, the AUC was estimated to be 0.70 (95% CI, 0.69 to 0.71) for STRATUS and Volpara, compared with the AUC of 0.68 (95% CI, 0.67 to 0.69) under BOADICEA v7 (2.9% increase, *P* value < 10^−4^). In contrast, the age-specific BIRADS implementation only improved the AUC slightly, in comparison with BOADICEA v7.

## DISCUSSION

BOADICEA (as implemented in the CanRisk tool) has been endorsed by several clinical management guidelines worldwide to guide decisions on screening or prevention for at-risk individuals.^39-44^ Here, we extended it by incorporating continuous MD. This led to an increased range of predicted BC risks, improved model discrimination, and reclassification. The new model was well calibrated in predicting risks across different risk categories.

The estimated relative risks, for women whose PMD/PVD is 1SD higher than the population average (Data Supplement and Table S9), were consistent with published ones.^16,23,30,45,46^ The 1%-2% attenuation of the association between BC and PMD/PVD when adjusting for PGS was also consistent with published estimates.^16^ Finally, the OPERA values of our estimates (which here coincide with the estimated HRs per SD) are in line with published OPERA values on continuous MD.^26,47,48^

Other models incorporate continuous MD data. For example, the Tyrer-Cuzick model considers Volpara PVD.^17^ However, unlike BOADICEA, where MD directly influences the age-specific BC incidence in the model, Tyrer-Cuzick includes Volpara as a postprediction adjustment to the predicted risk.^49-51^ Validation studies of the Tyrer-Cuzick model using Volpara reported AUCs of 0.61-0.62 for the 10-year BC risk (using FH and QRFs, but no genetic data).^50,51^ Other models have been developed to include STRATUS PMD^16,52^ in combination with other AI-based image features; validation studies of those models (in KARMA) reported AUCs of 0.77 for the 2-year BC risk^16^ and 0.75-0.65 for the 1- to 10-year BC risks.^52,53^

BMI and MD are not independent variables. However, in practice, they might not both be available. To allow for this flexibility, a key aspect of the extended BOADICEA model is that it uses two separate sets of parameters for STRATUS and Volpara, depending on whether BMI is available. A similar approach is used when PGS data are available, where a somewhat attenuated effect size for MD is assumed based on our estimates (and expected, due to the correlation between MD and PGS).

A key strength of the work is the use of the KARMA cohort, which includes information on all the risk factors used in BOADICEA. This has enabled us to estimate the associations of MD with BC risk while adjusting for all other risk factors included in BOADICEA.

Our approach of first calculating standardized residuals of the continuous MD (Data Supplement) ensures that the process can be applied to any combination of age, BMI, and PMD/PVD. In addition to the gain in discrimination, a significant advantage of using continuous rather than categorical density is that, by regressing out age, MD can be treated as a fixed covariate, removing the complexities of modeling a time-dependent covariate. The methodology presented here is flexible and can be used, in principle, for any continuous measure that can be suitably standardized. This should allow BOADICEA to be extended to incorporate not only other measures of MD but also other image-based risk measures that are rapidly emerging through novel machine learning approaches.^54,55^

The study has also some limitations. About 40% of the KARMA participants had missing data on some covariates, so imputation was used in both the development and validation stages. Only about 25% of its participant had PGS data, so a weighted cohort approach was used for analyses involving PGS. Data sets with more complete genetic data could provide more precise estimates on the associations between MD measurements and BC risk after adjusting for PGS. Also, we did not consider continuous MD measures in the context of pathogenic variants (PV) in risk genes: BOADICEA considers PVs, but the data on PVs in KARMA were too sparse to evaluate the model in PV carriers; larger cohorts with PV and MD data are required.

The model validation was performed using a testing subset of the cohort that was not used in the HR estimation; although this subset was independent, it still came from the same population (hence, similar characteristics) of the training data set (Data Supplement, Tables S4 and S5). The performance of the model may therefore have been overestimated, and additional studies are required to assess the model performance in completely different cohorts. On the other hand, the KARMA data set only reported computationally derived categorical BIRADS scores; this possibly masked the extent of the model improvement obtained using continuous MD measures (compared with manually assigned BIRADS scores).

Moreover, since the youngest women in the KARMA cohort were 40 years old at entry, the model was not directly validated for younger ages. Finally, the KARMA cohort is largely composed of White European women; therefore, it less clear whether the estimated parameters would be directly applicable to other ethnic groups. Published data suggest good agreement in the BIRADS distributions for White, Black, and mixed ethnicities; however, the BIRADS distributions for women of Asian ethnicity are markedly different.^8,56^ Therefore, model parameters for continuous MD should likely be recalculated, and the model re-evaluated, for Asians.

In conclusion, the updated BOADICEA model (v7.2), incorporating continuous MD measurements alongside the BIRADS classification, is available online through CanRisk.^10^ Although the routine availability of continuous MD measures varies by country (and most screening guidelines still refer to BIRADS categories^57^), the updated model allows for additional flexibility, providing a means of facilitating population-scalable approaches to incorporating MD into routine BC risk assessment.

## Data Availability

A data sharing statement provided by the authors is available with this article at DOI https://doi.org/10.1200/PO-25-00203. Data from the KARMA study are available upon request from Karolinska Institutet, through the Material Transfer Agreement form available at https://karmastudy.org/contact/data-access/.
